# Intrauterine Transmission of *Anaplasma phagocytophilum* in Persistently Infected Lambs

**DOI:** 10.3390/vetsci5010025

**Published:** 2018-02-28

**Authors:** Snorre Stuen, Wenche Okstad, Anne Mette Sagen

**Affiliations:** Section for Small Ruminant Research, Department of Production Animal Clinical Sciences, Norwegian University of Life Sciences, N-4325 Sandnes, Norway; wenche.okstad@nmbu.no (W.O.); amcsagen@hotmail.com (A.M.S.)

**Keywords:** *Anaplasma phagocytophilum*, persistence, intrauterine transmission, sheep

## Abstract

*Anaplasma phagocytophilum,* which causes the disease tick-borne fever (TBF), is the most important tick-borne pathogen in European animals. TBF may contribute to severe welfare challenges and economic losses in the Norwegian sheep industry. The bacterium causes a persistent infection in sheep and several other animal species. The objective of this study was to investigate whether intrauterine transmission occurs in persistently infected sheep. The study included thirteen 5–6-month-old unmated ewes, of which twelve were experimentally infected with *A. phagocytophilum* (GenBank acc. no. M73220). Four to six weeks later, all ewes were mated, and nine became pregnant. Blood samples were collected from these ewes and their offspring. If the lamb died, tissue samples were collected. The samples were analyzed with real-time PCR (qPCR) targeting the *msp2* gene. PCR-positive samples were further analyzed by semi-nested PCR and 16S rDNA sequencing. A total of 20 lambs were born, of which six died within two days. Six newborn lambs (30%) were PCR-positive (qPCR), of which one was verified by 16S rDNA sequencing. The present study indicates that intrauterine transmission of *A. phagocytophilum* in persistently infected sheep may occur. The importance of these findings for the epidemiology of *A. phagocytophilum* needs to be further investigated.

## 1. Introduction 

*Anaplasma phagocytophilum* (formerly *Ehrlichia phagocytophila*) may cause disease in several mammalian species including humans. In ruminants, the disease is named tick-borne fever (TBF) [1,2]. The infection is common on *Ixodes ricinus*-infested pastures in Europe [3] and not only represents a welfare challenge but also may cause severe economic losses. TBF is widespread in Norway and for decades has been one of the main scourges for the sheep industry in the coastal areas. More than 300,000 lambs are estimated to be infected each year. Diagnosis of TBF is normally based on blood smear microscopy, PCR-analyses, and serology [2,4]. 

*A. phagocytophilum* may cause persistent infection in several animal species [2], and sheep harbour the organism in their peripheral blood for months or even years [5,6]. This has to be considered when purchasing animals that have been on tick-infested pastures. There is no evidence of transovarial transmittance of the bacterium in *I. ricinus* ticks [7,8], so larvae become infected by ingesting blood from an infectious host or by co-feeding transmission from another infected tick. Maintenance in nature therefore depends on the availability of mammalian hosts [9]. The present study investigates if persistently infected sheep can transmit *A. phagocytophilum* to their offspring during pregnancy, i.e., if intrauterine infection occurs. This mode of pathogen transmission may have important epidemiological consequences. 

## 2. Materials and Methods

Thirteen 5–6-month-old unmated ewes of the Norwegian white breed (NKS) were used. Twelve ewes were inoculated intravenously with 0.4 mL of a whole blood dimethyl sulphoxide stabilate of an *A. phagocytophilum* strain (GenBank acc. no. M73220), containing approximately 1 × 10^6^ infected cells/mL and originally isolated from a local sheep flock [10]. One ewe was left as uninfected control. The animals had not previously been on a tick-infected pasture and were housed indoors during the entire experimental period. The study was approved by the National Animal Research Authority (Norway).

Rectal temperature was measured daily, and blood samples (EDTA) were collected regularly for the first month after inoculation. Haematology was performed using the ADVIA 120 Haematology System (Bayer, Tarrytown, NY, USA). Thereafter, blood samples, including serum samples, were obtained monthly. The ewes were mated 4–6 weeks post-inoculation (p.i.) with *A. phagocytophilum*. After delivery the following spring, blood samples (EDTA) and serum samples from the newborn lambs were obtained on the day of birth (day 0) and thereafter on days 3, 7, 14, 28, and 42. If the lamb died, tissue samples were collected, such as brain, lung, heart, spleen, liver, and heart blood. In addition, birth fluids and colostrum were collected at birth. The newborn lambs from the uninfected ewe were used as controls. All samples were stored at −20 °C before analysis.

### 2.1. DNA Extraction from Blood, Fetal Fluid, and Colostrum 

Samples were thawed at room temperature, and 200 µl was used for extraction. DNA was extracted with the MagNA Pure LC Instrument DNA Isolation Kit I (Roche Diagnostics, Mannheim, Germany) according to the manufacturer’s and instrument’s protocol. The DNA I blood cells high-performance program was used. The eluted DNA samples were stored at −20 °C. 

### 2.2. DNA Extraction from Tissue 

DNA from the tissue samples was extracted with the use of the MagNA Pure LC Instrument DNA Isolation Kit II (Roche Diagnostics, Mannheim, Germany) according to the manufacturer’s and the machine’s protocol. The DNA II tissue external Proteinase K program was used. Twenty-five milligrams of tissue was weighed and homogenized in 160 µL of tissue lysis buffer and 40 µL of Proteinase K. The samples were incubated at 55 °C overnight. One hundred microliters of the homogenized tissue samples was used for extraction, and the eluted DNA samples were stored at −20 °C.

### 2.3. Real-Time PCR for the Identification of Positive Samples, Targeting the Msp2 (P44) Gene 

The qPCR analysis was performed in a Light Cycler 480 Instrument (Roche Diagnostics, Mannheim, Germany) as described in previous studies [11]. Briefly, the reaction mix per sample consisted of forward primer ApMSP2252 (ACAGTCCAGCGTTTAGCAAGA), reverse primer ApMSP2459 (GCACCACCAATACCATAACCA), RNAse free H_2_O, Light Cycler 480 DNA SYBR Green I Master mix, Mannheim, Germany and template. The extracted DNA samples and reaction mix were loaded onto a 96-well white plate. The primers (TIB MOLBIOL Syntheselabor GmbH, Berlin, Germany) amplify a product of 208 bp covering the conserved N-terminal region of the expression site for the *msp2 (p44)* gene. Negative and positive controls were included. The positive control was obtained from an inoculated animal during the acute course of infection, whereas the negative control was a reaction mix without any added substance. Samples with a crossing point (Cp) ≤ 40 were validated by T_m_ analysis in a range of 82–83 °C [12]. All samples with an indication of *A. phagocytophilum* DNA were further analyzed by semi-nested PCR and sequenced for verification. 

### 2.4. Semi-Nested PCR and Sequencing of the 16S rRNA Gene 

Semi-nested PCR was carried out in a PTC-200 instrument (MJ Research, St Bruno, QC, Canada) as earlier described [13]. Briefly, the reaction was performed with an outer set of primers using the forward primer16S-F5 (5’AGTTTGATCATGGTTCAGA) and the reverse primer ANA-R4B (5’CGAACAACGCTTGC) for the amplification of a 507 bp fragment of *rrs* (16S rDNA gene) in *A. phagocytophilum*. Thereafter, the products were used in a second reaction with the same forward primer (16S-F5) and the new reverse primer ANA-R5 (5’TCCTCTAGACCAGCTATA) to amplify a 282 bp fragment. The amplicons from the semi-nested PCR were validated by gel electrophoresis on a 2% agarose gel. Positive PCR products were sequenced directly using BigDye Terminator cycle sequencing chemistry and capillary electrophoresis (ABI 310; Applied Biosystems, Foster, CA, USA), and *A. phagocytophilum* variants were detected by visual inspection of the chromatograms. The finished sequences were compared with databases by a BLAST (Basic Local Alignment Tool) search in NCBI’s webpage (http://blast.ncbi.nlm.nih.gov/Blast/). 

### 2.5. Serology 

Sera were analyzed using an indirect immunofluorescence antibody assay (IFA), to determine the antibody titre of an equine variant of *A. phagocytophilum* [14,15]. Briefly, two-fold dilutions of sera were added to slides precoated with *A. phagocytophilum* antigen (Protatec, St. Paul, MN, USA). Bound antibodies were visualized by fluorescein isothiocyanate (FITC)-conjugated rabbit-anti-sheep immunoglobulin (Cappel, Organon Teknika Corp., West Chester, PA, USA). Sera were screened for antibodies at dilution of 1:40. If positive, the serum was further diluted and retested. A titre of 1.6 (log_10_ reciprocal of 1:40) or more was considered positive. 

## 3. Results 

The unmated ewes infected with *A. phagocytophilum* reacted with a fever (≥40 °C), bacteraemia, and neutropenia during the next two weeks (Table 1). All were confirmed positive for this infection by PCR and gene sequencing (data not shown). Other clinical observations were dullness and reduced appetite for one or two days. No clinical or haematological reactions were recorded in the control lamb. All ewes were mated 4–6 weeks p.i., and nine became pregnant. In the following spring, a total of 20 lambs were born, of which six died within two days. Triplet lambs, which were delivered from one lame ewe that commenced parturition 14 days preterm, were weak at birth and died within one hour. Another dead lamb was stillborn. In addition, two lambs were weak-born and died within one day without any specific post-mortem diagnosis. No clinical signs in the other lambs were observed. 

The ewes were found to be PCR-positive at different occasions after the primary infection, especially during the period of delivery (data not shown). A total of 84 blood samples from newborn live lambs were investigated, and indication of an *A. phagocytophilum* infection (positive DNA) was only suspected in two lambs, which belonged to the same ewe. One of these lambs was found to be PCR-positive on two occasions: days 7 and 28 (Table 2). Birth fluids and colostrum were found to be negative by qPCR. In addition, a total of 65 tissue samples from six dead lambs were analyzed, and suspicion of an *A. phagocytophilum* infection was found in different tissues from four lambs (67%), of which three belonged to the same ewe (Table 3). In summary, three blood samples and twelve tissue samples from altogether six newborn lambs were positive by qPCR. Only one of these six lambs was found positive for *A. phagocytophilum* by semi-nested PCR and sequencing of the 16S rDNA gene (data not shown). 

In addition, antibodies against *A. phagocytophilum* were detected in all newborn live lambs, except for the precolostral samples (Table 4).

## 4. Discussion

The present study indicates that intrauterine transmission of *A. phagocytophilum* in persistently infected sheep may occur, as demonstrated by the fact that six of 20 (30%) newborn lambs were PCR-positive (qPCR) for *Anaplasma* DNA. However, further investigation is needed in order to confirm a viable *A. phagocytophilum* infection in these newborn lambs.

Several microorganisms have evolved effective ways to escape the host’s immune system and can in this way persist in the host [16]. As already mentioned, *A. phagocytophilum* may cause a persistent infection in different animal species, of which sheep can remain infected 25 months after the initial infection [5]. The cause of persistence in *A. phagocytophilum*-infected sheep is not known, but antigenic variation of major surface proteins has been proposed to be the key feature to allow persistence [17,18]. *A. phagocytophilum* shows a cyclic bacteraemia in infected sheep, characterized by varying numbers of organisms in the blood [19]. However, it may be difficult to verify an infection, especially since the circulating bacterial number declines during the persistence period, and sheep can be seropositive for several months after the primary infection [4,19,20]. 

In the present study, all inoculated lambs reacted with a fever typical for TBF [21]. Thereafter, the lambs remained infected for months, as earlier observed using the same *A. phagocytophilum* variant [19]. In a previous study, experimentally infected lambs became persistently infected for at least six months. Abortions were not observed during this study, despite earlier studies showing that acute infection during pregnancy may cause abortion [4,22,23,24]. However, only eight of the twelve persistently infected lambs were found pregnant. Whether ewes became barren as a consequence of *A. phagocytophilum* infection has to be further investigated. 

*A. phagocytophilum* are usually transmitted by ticks, but other pathways have been reported. For instance, human infection has been recorded by blood transfusion [25] and after the butchering of infected animals (via skin lesions) [26]. However, the latter pathway has not been properly verified. Nosocomial transmission has also been suggested [27]. Incidents via perinatal transmission in humans have been recorded, but the route of infection was difficult to determine [28,29]. In addition, intrauterine transmission of *A. phagocytophilum* has been observed in cattle during the acute phase of the infection [30,31]. It may be mentioned that the closely related bacterium *A. marginal,* has been reported to be transmitted to calves via placenta in chronically infected cows [32]. 

In the acute phase of the infection, a human isolate (NY-18) of *A. phagocytophilum* was transmitted to one newborn lamb during pregnancy [33], however there is no evidence yet for intrauterine transmission in persistently infected sheep. In the present study, six newborn lambs were PCR-positive (30%), of which one was confirmed by gene sequencing. The reason for this low number of verified cases may be due to a low amount of *A. phagocytophilum* DNA in the original samples. The present result indicates that qPCR is more sensitive than semi-nested PCR in the detection of *A. phagocytophilum*, as has earlier been reported [34].

Three pregnant animals had an indication of blood infection both right before and after delivery, and the newborn lambs from these ewes were all PCR-positive. Individual variations in the clinical and immunological response and cyclic bacteraemia have been previously observed [4,19]. However, only a limited number of samples were obtained from the pregnant ewes, so any relation between *A. phagocytophilum* cycling and intrauterine transmission has to be further elucidated. 

Of the six PCR-positive newborn lambs, all except two lambs died (Table 2 and Table 3). Whether an *A. phagocytophilum* infection causes stillborn and weak-born lambs is an open question. As already mentioned, three weak-born lambs were born 14 days before the estimated lambing time, which is beyond the expected time of survival after an early delivery [34]. 

In sheep, immunoglobulins are not normally transferred to the fetus via the placenta; a newborn lamb will therefore obtain maternal antibodies by ingesting colostrum during the first 48 hours [35]. Fetal lambs can synthesize immunoglobulins after 70 days of gestation and therefore be immunological competent during the last period of pregnancy [36], whereas infection before 70 days may lead to tolerance [37]. In the present study, serological tests of the precolostral blood were negative, indicating that *A. phagocytophilum-*positive lambs may have been infected before day 70. However, this has to be confirmed in future studies. Newborn lambs could have been infected by *A. phagocytophilum* by ingesting fetal fluid and colostrum, but both fluids were PCR-negative in the pregnant ewes. In addition, three of the PCR-positive newborn lambs died before any colostral intake. 

The variant of *A. phagocytophilum* in the present study has been previously used in different experimental trials [4]. However, several strains and variants of the bacterium in sheep have been characterized, which may cause different clinical manifestations [38]. Whether all variants of *A. phagocytophilum* affecting sheep cause intrauterine infection remains an open question.

## 5. Conclusions

In conclusion, persistently infected sheep may carry the pathogen from one grazing season to another. The present study indicates that the bacterium can be transmitted from ewe to offspring during pregnancy. However, only *A. phagocytophilum* DNA was detected. In order to confirm a viable *A. phagocytophilum* infection in newborn lambs, tick transmission or blood inoculation studies using susceptible lambs should be performed. Future studies will hopefully reveal if intrauterine transmission plays a role in the epidemiology of *A. phagocytophilum*.

## Figures and Tables

**Table 1:** Mean (±SD) values of clinical variables in 12 young, previously unmated ewes inoculated with *Anaplasma phagocytophilum*-infected blood.

Clinical Variables	Mean (±SD)
Incubation period (days)	3.8 ± 0.37
Maximum fever (°C)	41.62 ± 0.26
Duration of fever (days)	9.4 ± 3.93
Bacteraemia (day 5)	52.3 ± 12.3
Neutopenia on day 12 (g/L)	0.41 ± 0.12

**Table 2:** qPCR results of blood samples from triplet lambs (3a–3c) analyzed for *A. phagocytophilum*, from birth (D0) to day 42 (D42) (+ = positive; − = negative). Crossing point (Cp)-values in parenthesis.

Lamb	D0	D3	D7	D14	D28	D42
3a	−	−	−	−	−	−
3b	−	−	+ (36.0)	−	+ (37.0)	−
3c	−	−	+ (34.75)	−	−	−

**Table 3:** qPCR results of tissue samples from six dead lambs (5a–5c are triplets) analyzed for *A. phagocytophilum* infection (+ = positive; − = negative; na = not analyzed). Cp-values in parenthesis.

Lamb	5a	5b	5c	9b	10a	12c
Brain	−	−	−	−	+ (36.54)	−
Heart	−	−	+ (38.83)	−	+ (35.5)	−
Lung	+ (36.4)	−	−	−	−	−
Liver	−	+ (40.49)	−	−	−	−
Kidney	+ (36.3)	+ (37.8)	+ (37.6)	−	−	−
Spleen	+ (38.0)	+ (36.0)	+ (40.0)	−	+ (36.5)	−
Umbilicus	−	−	−	−	−	−
Abomasum	−	−	−	−	−	−
Blood	−	−	−	−	−	na
Urine	−	−	−	−	na	−

**Table 4:** Reciprocal indirect immunofluorescence antibody assay titres for *A. phagocytophilum* of 15 newborn lambs from birth (D0 before colostral intake) to day 42 (D42). A titre lower than 40 was considered negative (a, b, c indicate siblings; C = control lamb; na = not analyzed).

Lamb	D0	D3	D14	D42
3a	<40	320	160	<40
3b	<40	320	80	<40
3c	<40	640	320	40
7a	<40	2560	320	160
7b	<40	1280	320	80
8a	<40	640	320	40
9a	<40	640	320	<40
9b	<40	320	na	na
10b	<40	1280	320	160
10c	<40	640	160	40
12a	<40	1280	640	320
12b	<40	2560	1280	320
13a	<40	1280	640	320
C	<40	<40	<40	<40
C	<40	<40	<40	<40
